# Supplementary material for: Interaction mechanisms quantified from dynamical features of frog choruses
Source: R Soc Open Sci. 2020 Mar 18;7(3):191693. doi: 10.1098/rsos.191693 (PMC7137965; doi:10.1098/rsos.191693)
Supplement: Supplementary Information [file rsos191693supp1.pdf]

Supplementary Information: Interaction Mechanisms Quantified from Dynamical Features of Frog Choruses

Kaiichiro Ota[1,2], Ikkyu Aihara[3,*], Toshio Aoyagi[2,4]

1 Cybozu, Inc., Tokyo, Japan

2 JST CREST, Tokyo, Japan

3 Graduate School of Systems and Information Engineering, University of Tsukuba, Tsukuba, Japan

4 Graduate School of Informatics, Kyoto University, Kyoto, Japan

∗ E-mail: aihara@cs.tsukuba.ac.jp

Data accessibility

The empirical data of call timing is provided as files named as *Dataset1-26th-May-2008-CallTiming-Frog1-3.dat*, *Dataset2-16th-June-2008-CallTiming-Frog1-3.dat*, *Dataset3-17th-June-2008-CallTiming-Frog1-3.dat*, and *Dataset4-29th-May-2009-CallTiming-Frog1-3.dat*. For instance, the file *Dataset1-26th-May-2008-CallTiming-Frog1.dat* provides call timing (sec) of Frog 1 that was obtained from Dataset 1 carried out on 26th, May, 2008. The result of the model identification is summarized in Figure S1. Note that the result of Dataset 3 is also shown in Figure 3 of the main manuscript.

Terminology

We summarize terminology used in our manuscript. Tables S1, S2, and S3 provide information on terminology used in a phase oscillator model (Equation (4.2) in the main manuscript), that in the time differential equation of a phase difference (Equation (4.6) in the main manuscript), and that in the Fokker-Plank equation (Equation (4.10)) in the main manuscript), respectively. As for the explanation on the time differential equation of a phase difference, we also added Figure S2 to show representative cases of an equilibrium state and a critical state.

Leader-follower relationship

The definition of leader-follower relationship assumed in this study is explained in Figure S3.

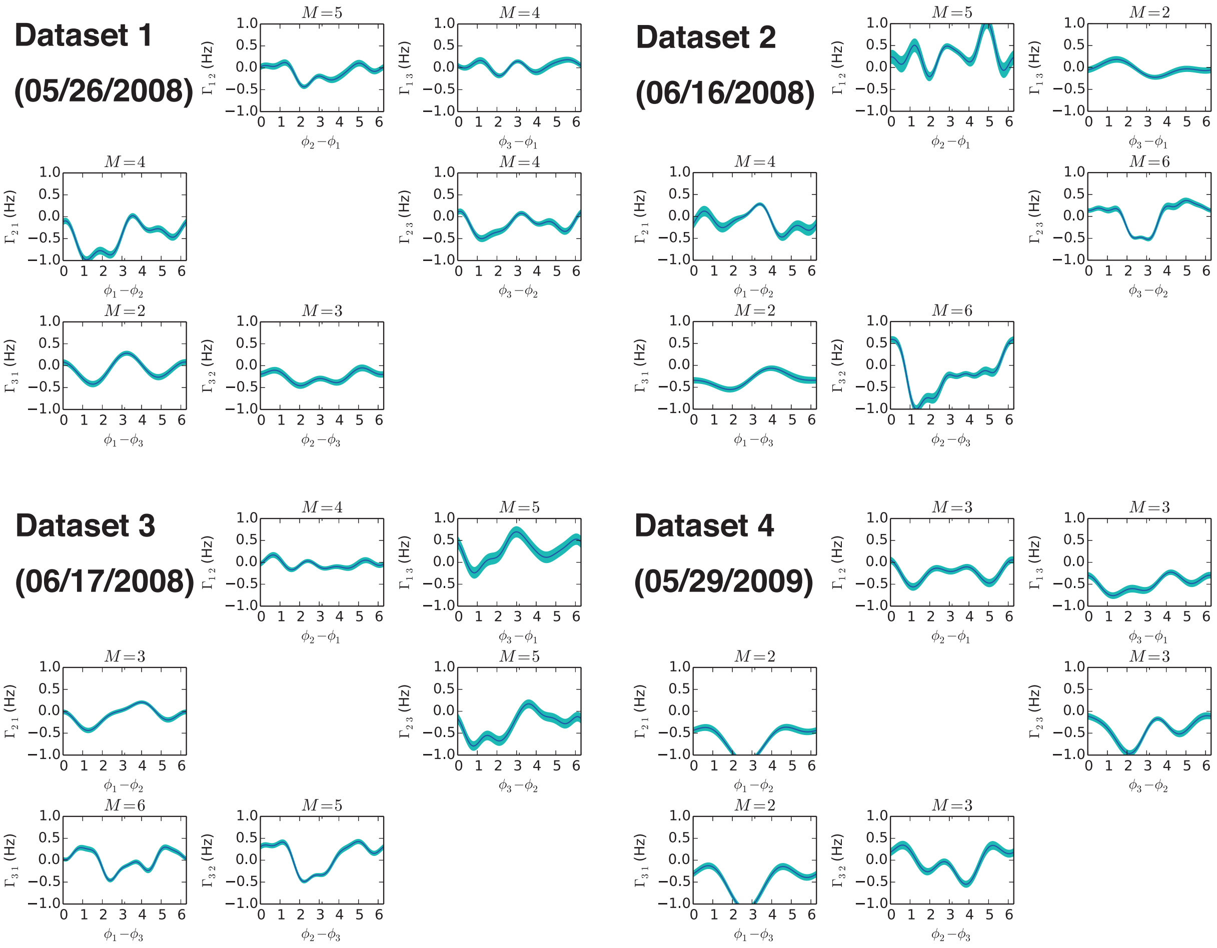

Figure S1. Unidirectional interaction terms of a phase oscillator model identified from the four datasets of call timing. Each dataset includes the call timing of three male Japanese tree frogs that were randomly captured on a different date at paddy fields at Kyoto University.

Subject	Symbol	Definition and brief explanation	Relevance to frog choruses
Phase	$\phi_n(t)$	This variable represents the phase of the nth frog at time t that ranges between 0 and 2π.	This variable represents call timing of the nth frog. Specifically, we assume that each frog produces a call when $\phi_n(t)$ hits 0.
Natural angular velocity	ω_n	This parameter represents the intrinsic angular velocity of the nth frog that takes a constant positive value.	This parameter represents the inter-call interval of the nth frog in an isolated situation. If the nth frog does not interact with other frogs and there is no noise, the phase $\phi_n(t)$ changes according to the equation $\frac{d\phi_n(t)}{dt} = \omega_n$. Subsequently, the phase $\phi_n(t)$ hits 0 at the interval of $2\pi/\omega_n$, corresponding to the situation in which the nth frog produces calls at the interval of $2\pi/\omega_n$.
Interaction term	$\Gamma_{nm}(\phi_n(t) - \phi_m(t))$	This term denotes the coupling function between the nth frog and the mth frog, and also is known to theoretically take the form of 2π-periodic function of the phase difference $\phi_n(t)-\phi_m(t)$ [1].	This term represents the acoustic interaction between the nth frog and the mth frog. In other words, we assume that the nth frog hears the calls of mth frogs, and controls his call timing according to this interaction term depending on the value of $\phi_n(t) - \phi_m(t)$.
Noise	$\xi_n(t)$	This term represents noise added to the nth oscillator. In this study, we assume $\xi_n(t)$ as white Gaussian noise given by $<\xi_n(t)\xi_n(s)>=\sigma_n\delta(t-s)$. Here, σ_n represents the magnitude of the noise.	This term represents small perturbation on periodic calling behavior of the nth frog. It is known that the inter-call interval of a male frog fluctuates even when he is isolated (in other words, when there is no other frog around him). To represent such small intrinsic fluctuation and unavoidable sources of uncertainty, for example, arising from observation error, we utilize the noise $\xi_n(t)$ that independently acts on each frog.

Table S1. Terminology used in a phase oscillator model (see Equation (4.2) in the main manuscript).

Subject	Symbol	Definition and brief explanation	Relevance to frog choruses
Phase difference	$\psi_{nm}(t) \equiv \phi_n(t) - \phi_m(t)$	This variable represents the difference of phases between the nth frog and the mth frog (i.e., $\phi_n(t) - \phi_m(t)$) that ranges from 0 to 2π.	This variable describes temporal structure in frog choruses. For example, $\psi_{nm} = \pi$ gives the situation in which the nth frog temporally alternates a call with that of the mth frog. In contrast, $\psi_{nm} = 0$ gives the situation in which the nth frog perfectly overlaps a call with that of the mth frog.
Equilibrium state (A stable equilibrium state)	ψ^*_{nm}	This value gives the phase difference satisfying two conditions of $G_{nm}(\psi^*_{nm}) = 0$ and $\partial G_{nm}(\psi_{nm})/\partial\psi_{nm}\|_{\psi_{nm}=\psi^*_{nm}} < 0$. If an equilibrium ψ^*_{nm} exists, the phase difference ψ_{nm} asymptotically converges to the value ψ^*_{nm}. An example of an equilibrium state is shown in Figure S2A.	An equilibrium state describes the behavior that a male frog produces calls at specific phase difference with those of another male. For example, $\psi^*_{nm} = \pi$ describes the situation in which the nth frog alternates calls with those of the mth frog quite robustly.
Critical State	ψ^{**}_{nm}	This value gives the phase difference between the nth frog and the mth frog satisfying the condition $\|G_{nm}(\psi^{**}_{nm})\| \simeq 0$. If a critical state ψ^{**}_{nm} exists, the phase difference stays around $\psi_{nm} = \psi^{**}_{nm}$ for a long time, and then intermittently leaves the point. An example of a critical state is shown in Figure S2B.	A critical state also describes the behavior that a male frog produces calls at specific phase difference with those of another male, while its stability is weaker than that of an equilibrium state. For example, $\psi^*_{nm} = \pi$ gives the situation in which the n frog alternates his calls with those of the mth frog for a long time, and then intermittently produces calls at unspecific phase difference with the mth frog.

Table S2. Terminology used in the time differential equation of a phase difference (see Equation (4.6) in the main manuscript).

Subject	Symbol	Definition and brief explanation	Relevance to frog choruses
Probability density function	$f(\psi_{nm}, t)$	This is the probability density function of a phase difference $\psi_{nm} \equiv \phi_n(t) - \phi_m(t)$ at time t.	This function is not directly relevant to chorusing frogs but can be utilized to numerically calculate the stationary distribution of the phase difference.
Stationary distribution of $f(\psi_{nm}, t)$	$\hat{f}(\psi_{nm})$	This represents the stationary distribution of the probability density function $f(\psi_{nm}, t)$. Technically, we calculated the time evolution of $f(\psi_{nm}, t)$ according to Equation (4.10) until it had converged, and then treated the converged $f(\psi_{nm}, t)$ as the stationary distribution $\hat{f}(\psi_{nm})$. Thus, the word "stationary" means that the distribution does not change any more in time and then it is stable.	This represents the distribution of a phase difference that is most expected to be realized by the identified phase oscillator model. For instance, if the stationary distribution has a peak around π, it is likely that the nth frog attempted to alternate calls with the mth frog at high probability.
Kullback-Leibler divergence	$D_{\mathrm{KL}}(\hat{f}\|\|u)$	This value defined by Equation (4.11) in the main manuscript quantifies the difference between the stationary distribution $\hat{f}(\psi_{nm})$ and uniform distribution $u(\psi_{nm})$.	We can utilize this value as the degree of attention paid among male frogs. For instance, larger $D_{\mathrm{KL}}(\hat{f}\|\|u)$ means that the stationary distribution $\hat{f}(\psi_{nm})$ shows an obvious peak, corresponding to the situation in which the nth frog responded to the calls of the mth frog consistently. In contrast, smaller $D_{\mathrm{KL}}(\hat{f}\|\|u)$ means that the stationary distribution $\hat{f}(\psi_{nm})$ is close to the uniform distribution $u(\psi_{nm})$, corresponding to the situation in which the nth frog produced calls at an unspecific interval with the mth frog.

Table S3. Terminology related to the Fokker-Plank equation (see Equation (4.10) in the main manuscript).

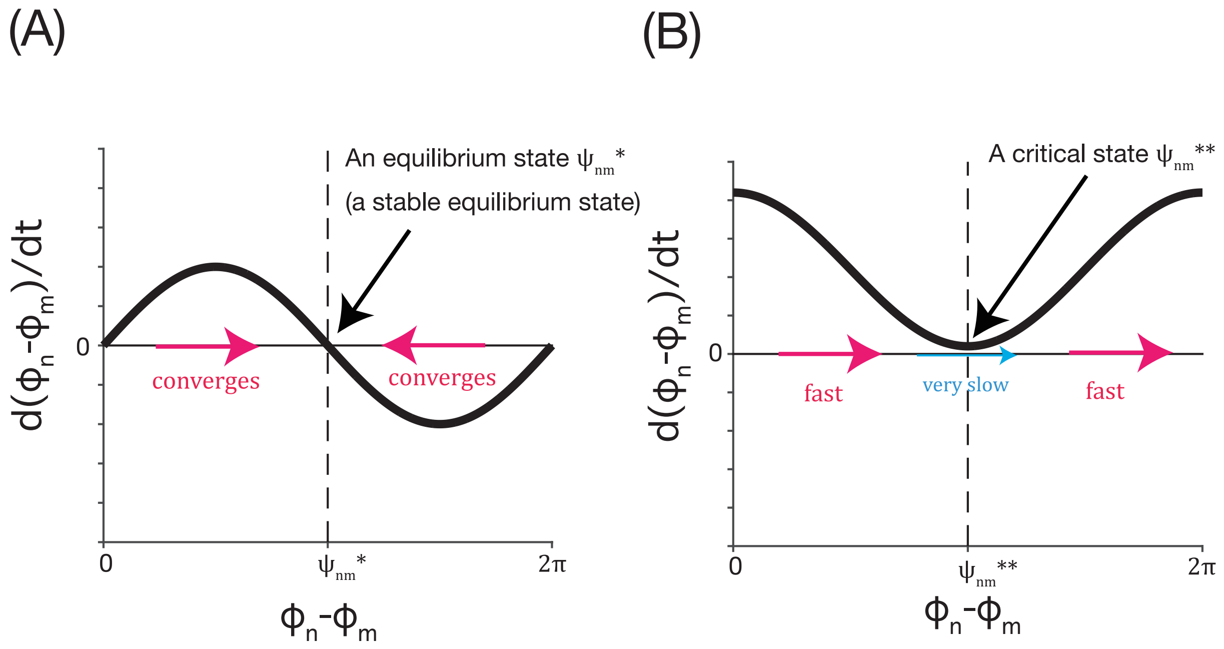

Figure S2. Schematic diagram on synchronized features in a phase oscillator model: (A) an equilibrium state ψ^*_{nm} and (B) a critical state ψ^{**}_{nm}. If an equilibrium state exists, a phase difference $\psi_{nm} \equiv \phi_n - \phi_m$ asymptotically converges to ψ^*_{nm}. In contrast, if a critical state exists, the phase difference ψ_{nm} remains around ψ^{**}_{nm} for a long time and then intermittently leaves the point.

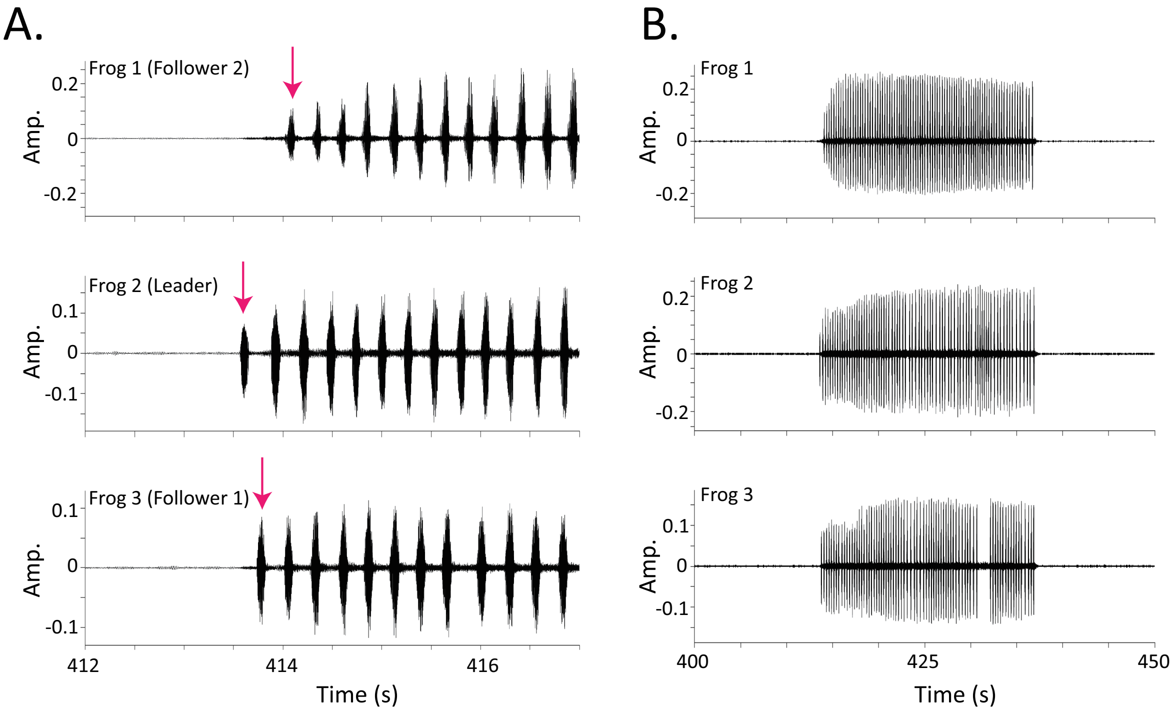

Figure S3. Leader-follower relationship of chorusing males. In this study, we define the leader, the 1st follower and the 2nd follower as the males that start calling first, second, and third within the same chorusing bout, respectively (Figure S3A). Male Japanese tree frogs tend to almost synchronize the start and end of their chorusing bouts with each other (Figure S3B) [2].

References

1. Kuramoto Y (1984) Chemical oscillations, waves, and turbulence. Berlin: Springer-Verlag.

2. Aihara I, Kominami D, Hirano Y, Murata M (2019) Mathematical modelling and application of frog choruses as an autonomous distributed communication system. Royal Society Open Science 6: 181117.
